# Development of Melioidosis Subunit Vaccines Using an Enzymatically Inactive Burkholderia pseudomallei AhpC

**DOI:** 10.1128/iai.00222-22

**Published:** 2022-07-11

**Authors:** Lindsey K. Schmidt, Caitlyn E. Orne, Teresa L. Shaffer, Shane M. Wilson, Nittaya Khakhum, Alfredo G. Torres, Paul J. Brett, Mary N. Burtnick

**Affiliations:** a Department of Microbiology and Immunology, University of Nevada, Reno School of Medicine, Reno, Nevada, USA; b Department of Microbiology and Immunology, University of Texas Medical Branch, Galveston, Texas, USA; c Department of Microbiology and Immunology, Faculty of Tropical Medicine, Mahidol University, Bangkok, Thailand; University of California San Diego School of Medicine

**Keywords:** *Burkholderia pseudomallei*, melioidosis, capsular polysaccharide, glycoconjugate, alkyl hydroperoxide reductase, subunit vaccine

## Abstract

Burkholderia pseudomallei, the causative agent of melioidosis, is a facultative intracellular, Gram-negative pathogen that is highly infectious via the respiratory route and can cause severe, debilitating, and often fatal diseases in humans and animals. At present, no licensed vaccines for immunization against this CDC Tier 1 select agent exist. Studies in our lab have previously demonstrated that subunit vaccine formulations consisting of a B. pseudomallei capsular polysaccharide (CPS)-based glycoconjugate (CPS-CRM197) combined with hemolysin-coregulated protein (Hcp1) provided C57BL/6 mice with high-level protection against an acute inhalational challenge of B. pseudomallei. In this study, we evaluated the immunogenicity and protective capacity of B. pseudomallei alkyl hydroperoxide reductase subunit C (AhpC) in combination with CPS-CRM197. AhpC is a peroxiredoxin involved in oxidative stress reduction and is a potential protective antigen. To facilitate our studies and maximize safety in animals, recombinant B. pseudomallei AhpC harboring an active site mutation (AhpC^C57G^) was expressed in Escherichia coli and purified using tandem nickel-cobalt affinity chromatography. Immunization of C57BL/6 mice with CPS-CRM197 combined with AhpC^C57G^ stimulated high-titer IgG responses against the CPS component of the glycoconjugate as well as stimulated high-titer IgG and robust interferon gamma (IFN-γ)-, interleukin-5 (IL-5)-, and IL-17-secreting T cell responses against AhpC^C57G^. When challenged via an inhalational route with a high dose (~27 50% lethal doses [LD_50_s]) of B. pseudomallei, 70% of the immunized mice survived 35 days postchallenge. Collectively, our findings demonstrate that AhpC^C57G^ is a potent activator of cellular and humoral immune responses and may be a promising candidate to include in future melioidosis subunit vaccines.

## INTRODUCTION

Melioidosis is an infectious disease of humans and animals caused by the Gram-negative bacillus Burkholderia pseudomallei (1). The clinical symptoms of melioidosis tend to be multifaceted, and disease may manifest as chronic or acute localized infections, acute pulmonary infections, or fulminating septicemias (2). Because of this, infections are often misdiagnosed and treated improperly (3, 4). Melioidosis is known to be endemic in 45 countries in areas ranging from Southeast Asia, South Asia, Australia, Africa, South America, and Central America to the Middle East. Current models predict an additional 34 countries where the disease is probably endemic but underreported, due to underdevelopment of microbiological facilities, lack of relevant clinical and laboratory expertise, and/or poor reporting systems (5). In 2015, the estimated total global burdens of melioidosis were ~165,000 cases and ~89,000 deaths annually, which is comparable to the mortality rate of measles and higher than the rates for leptospirosis and dengue fever (5). With the potential to impact over 79 countries, the global threat of melioidosis cannot be understated, and the prevention, diagnosis, and treatment of melioidosis are of paramount importance. In addition to its impact on public health, B. pseudomallei has the potential for misuse as a biothreat agent and is currently categorized as a Tier 1 select agent by the U.S. Centers for Disease Control and Prevention (CDC) (6).

B. pseudomallei is a motile, aerobic, facultative intracellular pathogen and a soil- and water-dwelling bacterium (7, 8). Acquisition of B. pseudomallei typically occurs through inhalation of aerosolized soil or water, percutaneous inoculation, or ingestion of contaminated food or water (4). The organism is naturally resistant to a variety of antibiotics, and there is high mortality associated with acute forms of the disease. Mortality rates can be as high as 50% in some regions of endemicity, which can increase to 80% if patients have one or more predisposing conditions, such as diabetes, alcoholism, chronic lung disease, chronic renal disease, thalassemia, or cancer, or are undergoing immunosuppressive therapy (9). B. pseudomallei infections can be difficult to treat and require intravenous ceftazidime or meropenem for at least 10 to 14 days, followed by 3 to 6 months of oral trimethoprim-sulfamethoxazole (10). Currently, there are no licensed vaccines available for immunization against melioidosis (6).

B. pseudomallei is a versatile pathogen that is capable of both extracellular and intracellular growth (11). Following infection, the organism can invade host cells, escape from phagosomes, survive within the cytosol, and spread from cell to cell in almost any tissue of the body (12–14). The B. pseudomallei genome is large (~7.2 Mb) and is estimated to carry approximately 5,855 coding sequences involved in a myriad of functions that enable survival in a range of environments (15, 16). The organism is resistant to host complement-mediated killing and has an abundance of virulence genes that are expressed following infection (15, 16). While surface polysaccharides such as the 6-deoxyheptan capsular polysaccharide (CPS) and the O-polysaccharide (OPS) component of lipopolysaccharide contribute to complement and serum resistance, B. pseudomallei also expresses a number of specialized secretion systems and detoxifying enzymes that facilitate its intracellular lifestyle (7). Due to the complex life cycle of B. pseudomallei and its tendency to cause persistent infections, an effective vaccine will need to stimulate both protective humoral and cellular immune responses.

Previous preclinical vaccine studies have explored the use of live attenuated bacteria, outer membrane vesicles, nanoparticle platforms, glycoconjugates, and multivalent subunit formulations with varied success (10, 17–26). Research in our lab focuses on the development of glycoconjugate-based subunit vaccines, an approach that is generally considered safe and utilizes a defined set of antigens, which allows for the determination of antigen-specific correlates of immunity (1, 6, 19, 24, 27). Previously, we demonstrated that mice immunized with CPS-based glycoconjugates along with recombinant *Burkholderia* proteins (e.g., LolC and TssM) were provided with significant protection against lethal challenges of B. pseudomallei (19, 24). Most recently, we found that when C57BL/6 mice were immunized with CPS conjugated to the diphtheria toxin mutant CRM197 (CPS-CRM197), combined with hemolysin-coregulated protein 1 (Hcp1) and adjuvanted with Alhydrogel and CpG, 100% of the mice were protected against a lethal inhalational challenge of B. pseudomallei, and 70% of the survivors had no culturable bacteria in their lungs, livers, or spleens (19). The immunized mice exhibited high-titer CPS-specific IgG responses that were opsonizing as well as high-titer Hcp1-specific IgG levels and robust interferon gamma (IFN-γ)-secreting T cell responses (19). Extending these findings, we have begun to explore the use of CPS-CRM197 in combination with different proteins that show promise as protective antigens to improve our existing vaccine formulation.

In the present study, we investigated the immunogenicity and protective capacity of B. pseudomallei alkyl hydroperoxide reductase subunit C (AhpC) when combined with CPS-CRM197. AhpC homologs are highly conserved across *Burkholderia* spp., and B. pseudomallei *ahpC* (BPSL2096) has been shown to be virtually invariant among clinical isolates (28). AhpC is a peroxiredoxin involved in protection against oxidative stress and is an immunodominant antigen that induces strong T cell-mediated responses in melioidosis patients (28–31). AhpC has been used as a serodiagnostic target and is considered a protective antigen and promising vaccine candidate (28, 29, 32). To facilitate our studies and maximize safety in animals, we purified a recombinant version of B. pseudomallei AhpC harboring an active site mutation (AhpC^C57G^). Results showed that a subunit vaccine consisting of CPS-CRM197 plus AhpC^C57G^ formulated with Alhydrogel and CpG provided high-level protection against a lethal aerosol challenge of B. pseudomallei in C57BL/6 mice.

## RESULTS

### Production of recombinant B. pseudomallei AhpC and AhpC^C57G^.

**FIG 1:**
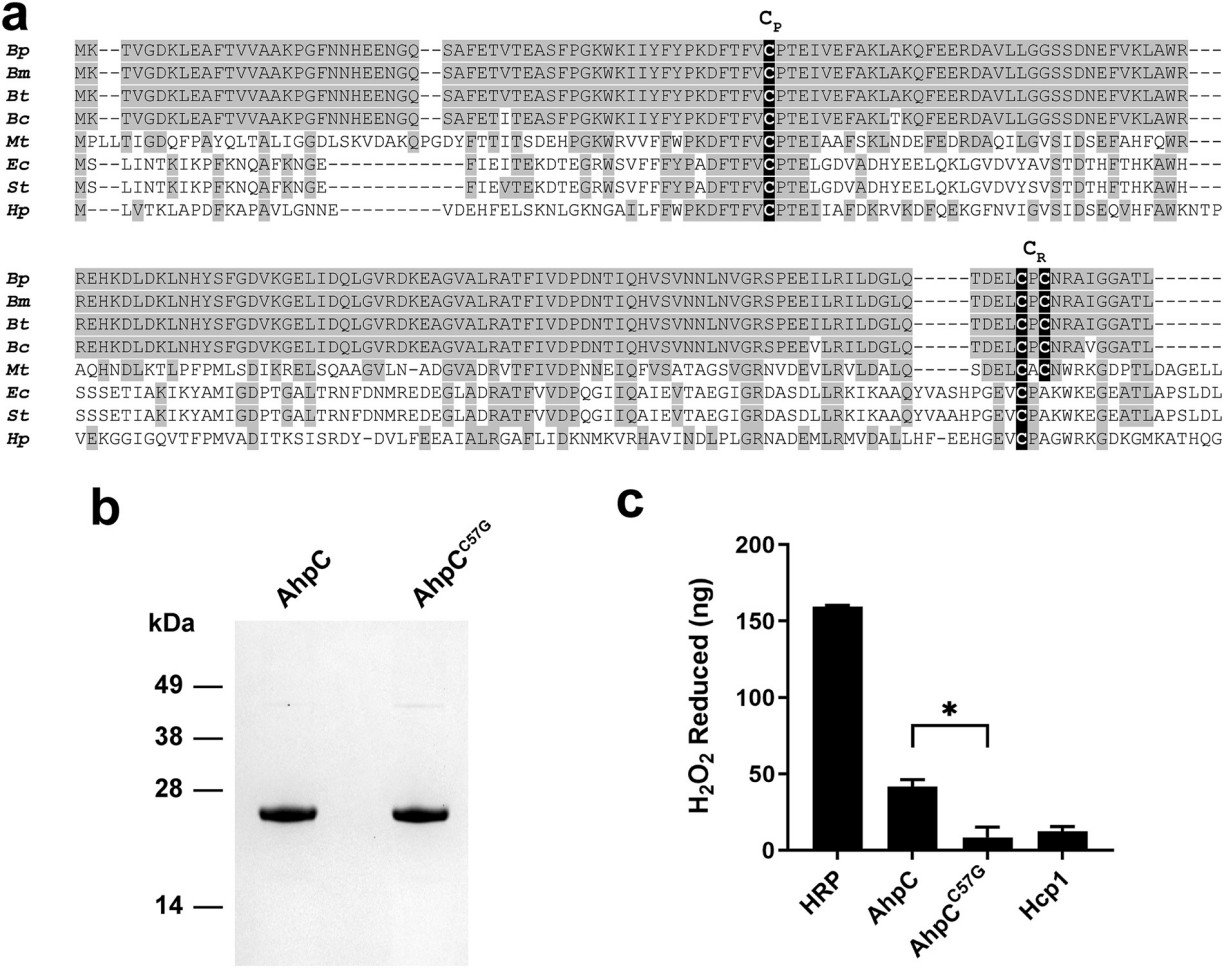
Physical and enzymatic analysis of B. pseudomallei AhpC and AhpC^C57G^ antigens. (a) Multiple-sequence alignment of B. pseudomallei (*Bp*), Burkholderia mallei (*Bm*), Burkholderia thailandensis (*Bt*), Burkholderia cepacia (*Bc*), Mycobacterium tuberculosis (*Mt*), Escherichia coli (*Ec*), Salmonella enterica serovar Typhimurium (*St*), and Helicobacter pylori (*Hp*) AhpC homologs. Conserved residues are highlighted in gray, while the N-terminal peroxidatic Cys (C_P_) residue and C-terminal resolving Cys (C_R_) residues are highlighted in black. (b) SDS-PAGE and SimplyBlue staining were used to assess the purity of the AhpC and AhpC^C57G^ preparations. Positions of the molecular weight standards (kilodaltons) are indicated on the left. (c) H_2_O_2_ reduction assays were used to assess the peroxidase activities associated with purified AhpC and AhpC^C57G^ antigens. Horseradish peroxidase (HRP) and hemolysin-coregulated protein 1 (Hcp1) were used as positive and negative controls, respectively. Bars represent the means ± standard deviation (SD) of results from individual assays conducted in duplicate. Values are representative of two independent experiments conducted on different days. *, *P* < 0.05.

AhpC enzymes are found in most prokaryotes and belong to a family of 2-Cys peroxiredoxins that catalyze the reduction of hydroperoxides for protection against oxygen radical damage (33). Previous studies have shown that there are two or three conserved cysteine residues critical for the enzymatic activity of AhpC: one at the N terminus (the peroxidatic cysteine, C_P_) and one or two at the C terminus (the resolving cysteine, C_R_) (34, 35). Zhang et al. recently characterized Burkholderia thailandensis AhpC and identified a C_P_ at position 57 and two C_R_ residues at positions 171 and 173 (31). As shown in Fig. 1a, the primary amino acid sequence of B. pseudomallei AhpC is 100% identical to that of B. thailandensis AhpC and harbors the same three cysteine residues (Cys57, Cys171, and Cys173). To mutate the active site of B. pseudomallei AhpC, the conserved N-terminal C_p_ (Cys57) was substituted with a glycine since it lacks a sulfur group and thus prevents the formation of the disulfide bonds that are necessary for AhpC activity (34). The resulting protein was designated AhpC^C57G^ (see Table 1). N-terminal His-tagged versions of recombinant AhpC and AhpC^C57G^ were expressed and purified from Escherichia coli using tandem nickel-cobalt chromatography, essentially as previously described for Hcp1 (36–38). The purity and structural integrity of AhpC and AhpC^C57G^ were assessed by SDS-PAGE (Fig. 1b). The endotoxin concentrations of the purified proteins were determined to be <21 endotoxin units (EU)/mg using a *Limulus* amoebocyte lysate (LAL) assay.

**TABLE 1:** Bacterial strains and plasmids used in this study

Strain or plasmid	Description^*a*^	Source or reference
Strains		
Escherichia coli		
TOP10	Lab strain for cloning and protein expression	Life Technologies
Burkholderia pseudomallei		
Bp82	Δ*purM* derivative of 1026b, select agent excluded strain	53
K96243	Wild type, clinical isolate from Thailand	16
Burkholderia thailandensis		
E555	Wild type, soil isolate from Cambodia, expresses CPS	54
BT2683	OPS-deficient derivative of E555, Δ*rmlD*	Unpublished data
Plasmids		
pBAD/HisA	Arabinose inducible, 6×His tag expression vector, Ap^r^	Life Technologies
pBADBmhcp1-6HisF	pBAD/HisA containing B. mallei *hcp1* (BPSS1498) with N-terminal His tag	37
pRR1000	pBAD/HisA containing B. pseudomallei *ahpC* (BPSL2096) with N-terminal His tag	This study
pLS1000	pCR2.1 containing B. pseudomallei *ahpC* with N-terminal His tag in which Cys codon (TGC) at position 57 in *ahpC* has been changed to Gly codon (GGC)	This study
pLS1001	pBAD/HisA containing B. pseudomallei *ahpC* with N-terminal His tag in which Cys codon (TGC) at position 57 in *ahpC* has been changed to Gly codon (GGC)	This study

a Ap^r^, ampicillin resistant.

To test the enzymatic activity of AhpC versus AhpC^C57G^, H_2_O_2_ reduction was measured using a peroxidase activity assay. Following incubation of the proteins with H_2_O_2_, residual H_2_O_2_ levels were measured using an iron and xylenol orange reagent that produces a measurable color change. As shown in Fig. 1c, AhpC reduced significantly more H_2_O_2_ than AhpC^C57G^. The level of AhpC^C57G^ activity was similar to that of Hcp1, which was used as a negative control. Horseradish peroxidase (HRP) was used as a positive control in the assay, and as expected, resulted in the largest amount of H_2_O_2_ being reduced. These results indicated that the N-terminal Cys57 residue is required for AhpC activity and acts as a C_P_ in the active site of the enzyme. Based on these findings, the enzymatically inactive protein AhpC^C57G^ was used in subsequent experiments.

### Identification of T cell-restricted epitopes in AhpC^C57G^ and T cell subtype reactivity against AhpC^C57G^.

**FIG 2:**
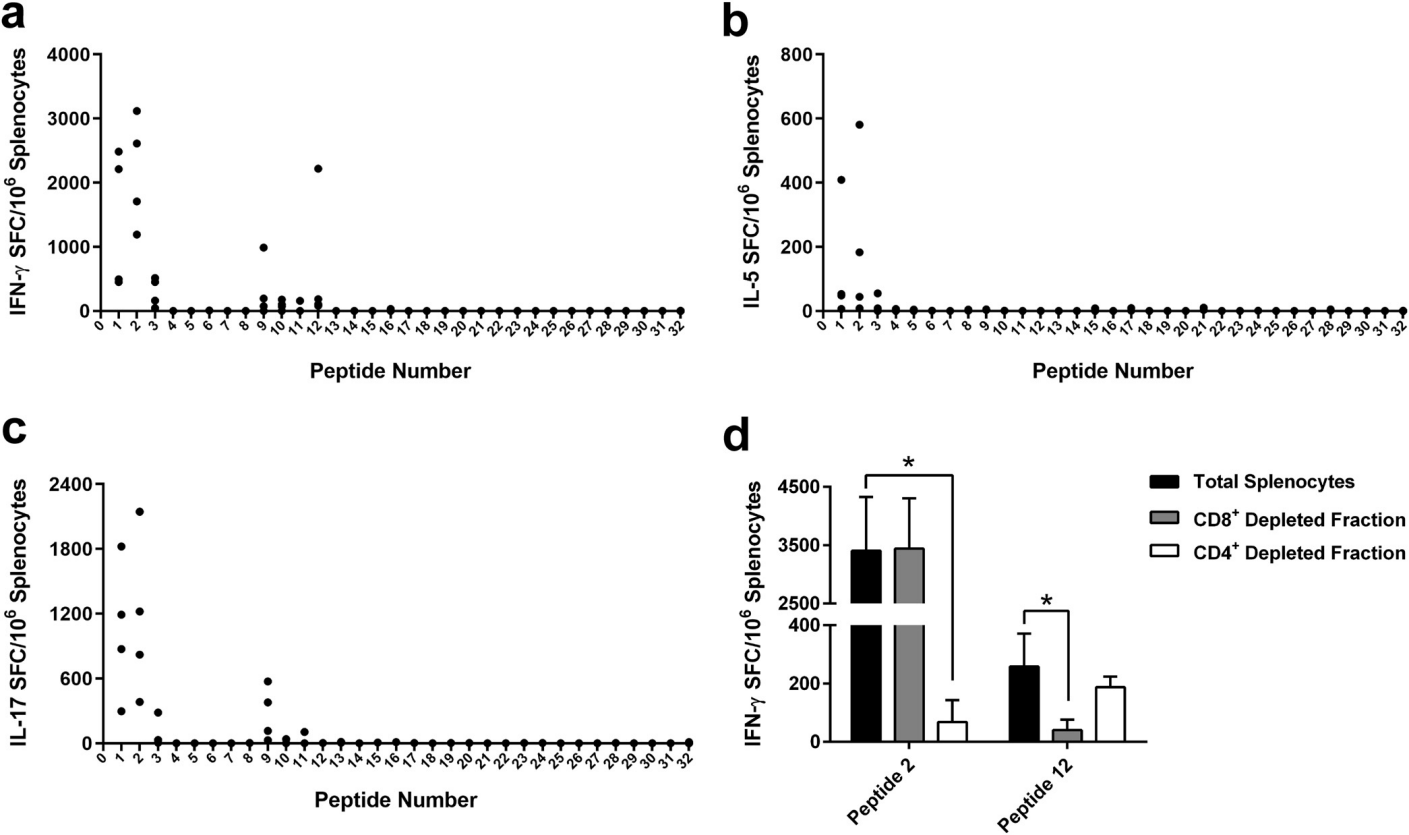
Identification and characterization of T cell-restricted epitopes associated with AhpC^C57G^. C57BL/6 mice (*n* = 12) were immunized on days 0, 21, and 35 with AhpC^C57G^. Spleens (*n* = 4 per analyte) were harvested on day 42, and (a) IFN-γ-, (b) IL-5-, and (c) IL-17-secreting T cell responses against an AhpC^C57G^ peptide library (15-mers overlapping by 10 amino acids) were quantitated by ELISpot. Black dots represent the means of assays conducted in duplicate for individual mice. SFC, spot-forming cells. (d) C57BL/6 mice (*n* = 3) were immunized on days 0, 21, and 35 with AhpC^C57G^. Spleens harvested on day 42 were combined and used to prepare three equal pools of cells. The pools were either depleted of CD8^+^ cells or CD4^+^ cells or left untreated, and IFN-γ-secreting T cell responses against peptide 2 and peptide 12 were quantitated by ELISpot. Bars represent the means ± SD from three individual experiments conducted in duplicate. *, *P* < 0.05.

Previous studies have shown that T cell responses to B. pseudomallei AhpC correlate with survival in melioidosis patients (28, 29). These responses were predominantly associated with CD4^+^ T cells, and two human-specific immunodominant peptides were identified using IFN-γ enzyme-linked immunosorbent spot (ELISpot) assays (29). To facilitate the analysis of T cell responses to AhpC^C57G^ in C57BL/6 mice, we conducted epitope mapping experiments using a peptide library consisting of 15-mers that overlapped by 10 amino acids (aa) (see Table 2 below). To generate immune splenocytes for use in these studies, mice were immunized three times with AhpC^C57G^ formulated with Alhydrogel and CpG. One week after the final boost, IFN-γ (Th1-like cytokine), IL-5 (Th2-like cytokine), and IL-17 (Th17-like cytokine) ELISpot assays were conducted using the individual peptides as recall antigens. Of the 35 peptides tested, peptides 1 and 2 stimulated robust IFN-γ-, IL-5-, and IL-17-secreting T cell responses, indicating a T cell-restricted epitope is located at the N terminus of AhpC^C57G^ (Fig. 2a to c). Additionally, peptides 9 to 11 stimulated both IFN-γ- and IL-17-secreting T cell responses, indicating that a second T cell epitope is present starting ~41 aa downstream from the N terminus. Interestingly, peptide 12 was found to stimulate only IFN-γ-secreting T cell responses, indicating the presence of a third unique T cell epitope in AhpC^C57G^.

**TABLE 2:** AhpC^C57G^ peptide library

Peptide no.	Amino acid sequence
1	KTVGDKLEAFTVVAA
2	KLEAFTVVAAKPGFN
3	TVVAAKPGFNNHEEN
4	KPGFNNHEENGQSAF
5	NHEENGQSAFETVTE
6	GQSAFETVTEASFPG
7	ETVTEASFPGKWKII
8	ASFPGKWKIIYFYPK
9	KWKIIYFYPKDFTFV
10	YFYPKDFTFVGPTEI
11	DFTFVGPTEIVEFAK
12	GPTEIVEFAKLAKQF
13	VEFAKLAKQFEERDA
14	LAKQFEERDAVLLGG
15	EERDAVLLGGSSDNE
16	VLLGGSSDNEFVKLA
17	SSDNEFVKLAWRREH
18	FVKLAWRREHKDLDK
19	WRREHKDLDKLNHYS
20	KDLDKLNHYSFGDVK
21	LNHYSFGDVKGELID
22	FGDVKGELIDQLGVR
23	GELIDQLGVRDKEAG
24	QLGVRDKEAGVALRA
25	DKEAGVALRATFIVD
26	VALRATFIVDPDNTI
27	TFIVDPDNTIQHVSV
28	PDNTIQHVSVNNLNV
29	QHVSVNNLNVGRSPE
30	NNLNVGRSPEEILRI
31	GRSPEEILRILDGLQ
32	EILRILDGLQTDELC
33	LDGLQTDELCPCNRA
34	TDELCPCNRAIGGAT
35	DELCPCNRAIGGATL

To determine the T cell population(s) that recognized AhpC-specific peptides 2 and 12, splenocytes from mice immunized with AhpC^C57G^ were pooled, depleted of either CD4^+^ or CD8^+^ T cells, and then analyzed using IFN-γ ELISpot assays (Fig. 2d). Flow cytometry was used to confirm efficient depletion of CD4^+^ or CD8^+^ in each of the samples (data not shown). The results showed that depletion of CD4^+^ T cells led to a significant reduction (*P* < 0.05) in the number of IFN-γ-secreting T cells that responded to AhpC^C57G^ and peptide 2. Conversely, depletion of CD8^+^ T cells resulted in significantly reduced numbers (*P* < 0.05) of IFN-γ-secreting T cells that responded to peptide 12. Taken together, these findings suggest that AhpC^C57G^ harbors epitopes that are recognized by both CD4^+^ and CD8^+^ T cells in C57BL/6 mice.

### Analysis of antibody responses raised against CPS-CRM197 and AhpC^C57G^.

**FIG 3:**
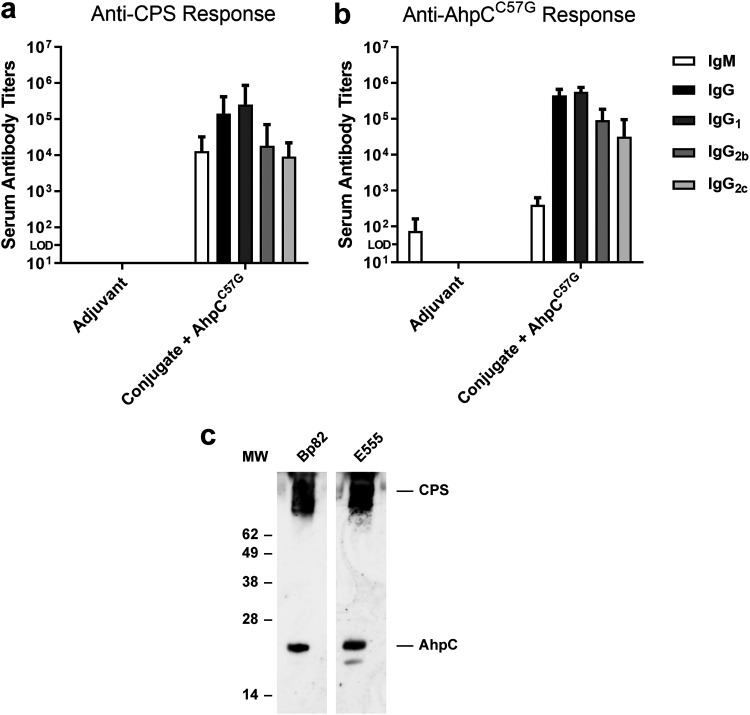
Characterization of antibody responses raised against CPS-CRM197 and AhpC^C57G^. C57BL/6 mice (*n* = 6 per group) were immunized on days 0, 21, and 35 with adjuvant only or CPS-CRM197 plus AhpC^C57G^. Immune serum samples were collected on day 42. ELISAs were used to quantitate serum IgM, IgG, IgG1, IgG2b, and IgG2c titers against (a) CPS and (b) AhpC^C57G^. Bars represent geometric means with 95% confidence interval (CI). Conjugate, CPS-CRM197; LOD, limit of detection. (c) Western immunoblot analysis of B. pseudomallei Bp82 and B. thailandensis E555 whole-cell lysates using pooled CPS-CRM197 plus AhpC^C57G^ immune serum. The positions of the molecular weight (MW) standards (kilodaltons) are indicated on the left.

CPS-based glycoconjugates in combination with recombinant *Burkholderia* proteins have previously been shown to stimulate robust antibody responses in mice (19, 24). To assess the immunogenicity of our vaccine formulation, groups of C57BL/6 mice were immunized with either adjuvant only or CPS-CRM197 plus AhpC^C57G^. One week after the final boost, serum was collected, and antigen-specific IgM and IgG titers were determined by enzyme-linked immunosorbent assay (ELISA). Results of these analyses showed that the vaccine antigens stimulated the production of high-titer IgM (mean endpoint titers of >10^4^) and total IgG (mean endpoint titers of >10^5^) responses against CPS (Fig. 3a). As expected, the combination of antigens also stimulated the production of IgM (mean endpoint titers of >10^2^) and high-titer total IgG (mean endpoint titers of >10^5^) responses against AhpC^C57G^ (Fig. 3b). Analysis of IgG1 and IgG2b levels in the immune serum samples revealed that relatively balanced Th2/Th1-type responses were generated against both antigens (Fig. 3a and b). Additionally, Western immunoblotting was conducted using whole-cell lysates of B. pseudomallei Bp82 and B. thailandensis E555 and demonstrated that the CPS- and AhpC^C57G^-specific antibodies also reacted with native B. pseudomallei and B. thailandensis antigens (Fig. 3c).

**FIG 4:**
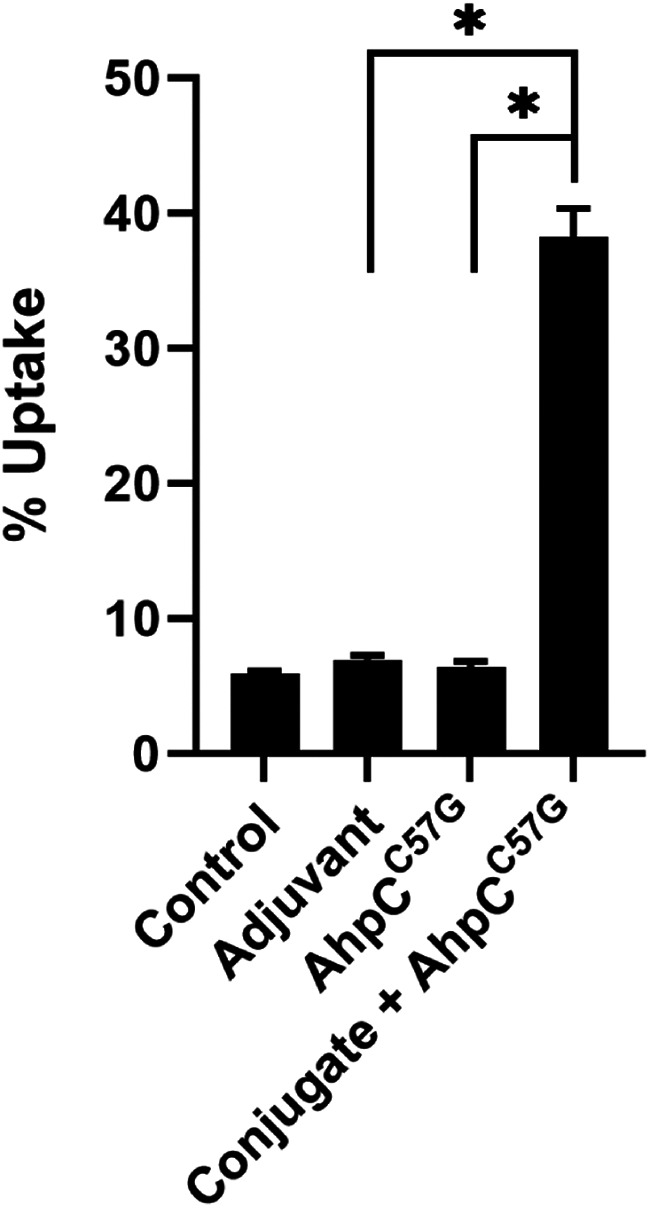
Functional analysis of antibody responses raised against CPS-CRM197 and AhpC^C57G^. C57BL/6 mice (*n* = 4 to 6 per group) were immunized on days 0, 21, and 35 with adjuvant only, AhpC^C57G^, or CPS-CRM197 plus AhpC^C57G^. Immune serum samples were collected on day 42. B. thailandensis E555 was incubated with medium only (no-serum control), pooled HI adjuvant-only immune serum, pooled HI AhpC^C57G^-only immune serum, and pooled HI conjugate-plus-AhpC^C57G^ immune serum. Following incubation for 1 h, opsonized bacteria were added to RAW 264.7 murine macrophage monolayers. Uptake was quantitated at 3 h postinfection. Bars represent the means ± SD of results from three individual assays conducted in triplicate. Values are representative of three independent experiments conducted on different days. *, *P* < 0.05.

To assess the functionality of the CPS-CRM197 and protein-specific antibodies, opsonophagocytosis assays were conducted. Since B. thailandensis E555 is avirulent, possesses the same 6-deoxyheptan CPS and AhpC as B. pseudomallei, and behaves similarly in RAW 264.7 mouse macrophages, this strain was used in opsonophagocytosis assays to eliminate the need for these studies to be conducted using biosafety level 3 (BSL3) containment (39). As shown in Fig. 4, preincubation of B. thailandensis E555 with heat-inactivated (HI) pooled antiserum from mice immunized with CPS-CRM197 plus AhpC^C57G^ significantly enhanced bacterial uptake (>5-fold) into RAW 264.7 cells compared to serum from nonconjugate controls. As expected, serum obtained from mice immunized with adjuvant only and AhpC^C57G^ exhibited no differences in uptake in comparison to the medium-only controls. Collectively, these results demonstrate that the conjugate material used in our vaccine formulation was capable of stimulating opsonizing antibody responses in C57BL/6 mice.

### Analysis of cellular immune responses raised against CPS-CRM197 and AhpC^C57G^.

**FIG 5:**
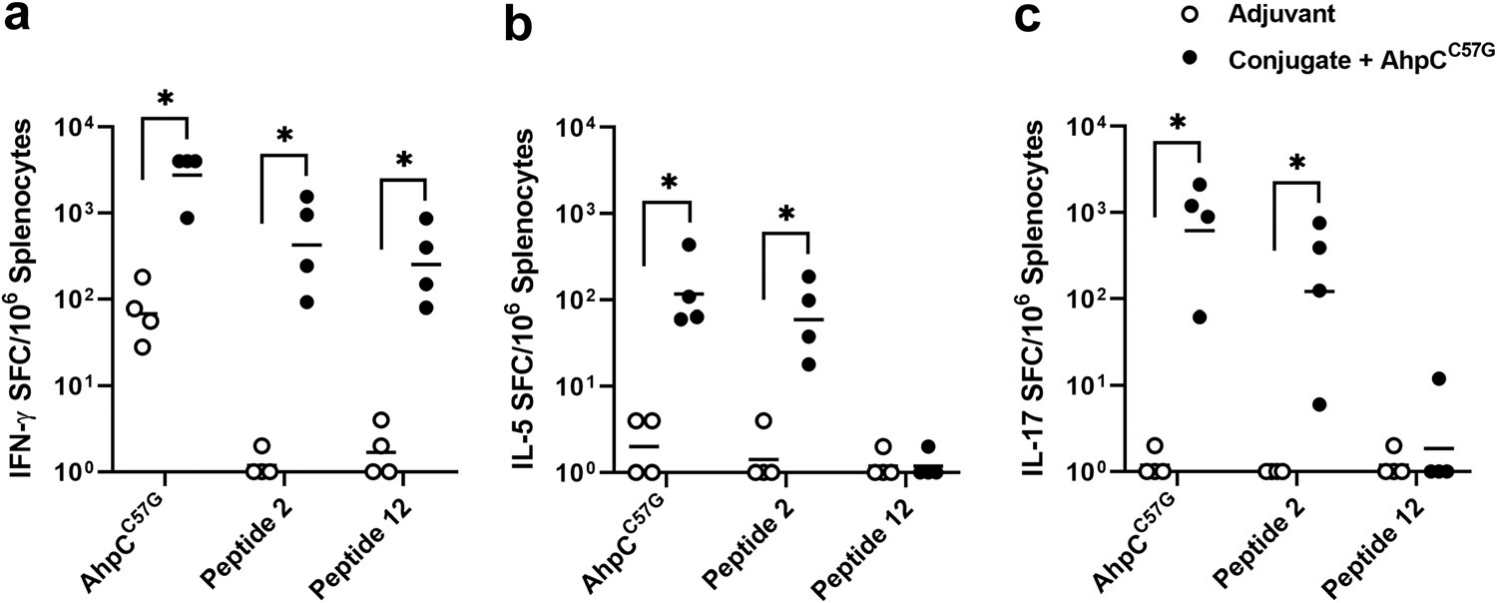
Characterization of cellular immune responses raised against AhpC^C57G^. C57BL/6 mice (*n* = 4 per group) were immunized on days 0, 21, and 35 with CPS-CRM197 plus AhpC^C57G^. Spleens were harvested on day 42, and (a) IFN-γ-, (b) IL-5-, and (c) IL-17-secreting T cell responses against AhpC^C57G^, peptide 2, and peptide 12 were quantitated by ELISpot. White and black dots represent the means of assays conducted in duplicate for individual mice. Black bars represent geometric means for each group. *, (*P* < 0.05).

Tapia et al. have previously demonstrated that immunity provided by multicomponent gold nanoparticle vaccines is associated with mixed Th1 and Th17 responses in a mouse model of melioidosis (25). It is also well established that IFN-γ-secreting T cell responses against various *Burkholderia* proteins correlate with survival of melioidosis patients (29, 40). Of note, AhpC has been shown to elicit some of the strongest T cell responses in survivors compared to other immunoreactive B. pseudomallei proteins (28, 29). To assess the AhpC-specific T cell responses following immunization of C57BL/6 mice with CPS-CRM197 plus AhpC^C57G^, single-cell splenocyte suspensions were prepared, restimulated with AhpC^C57G^, peptide 2, and peptide 12, and then analyzed using IFN-γ, IL-5, and IL-17 ELISpot assays to assess Th1-, Th2-, and Th17-like cellular immune responses, respectively. As shown in Fig. 5a, all mice exhibited robust IFN-γ-secreting T cell responses to all stimuli in comparison to the adjuvant-only control. In addition, AhpC^C57G^ and peptide 2, but not peptide 12, stimulated robust IL-5- and IL-17-secreting T cell responses in comparison to the adjuvant-only controls (Fig. 5b and c). These findings are consistent with the results of our peptide mapping experiments and demonstrate that the vaccine formulation CPS-CRM197 plus AhpC^C57G^ stimulates robust IFN-γ-, IL-5-, and IL-17-secreting T cell responses in C57BL/6 mice.

### Evaluation of the protective capacity of CPS-CRM197 plus AhpC^C57G^ in a mouse challenge study.

**FIG 6:**
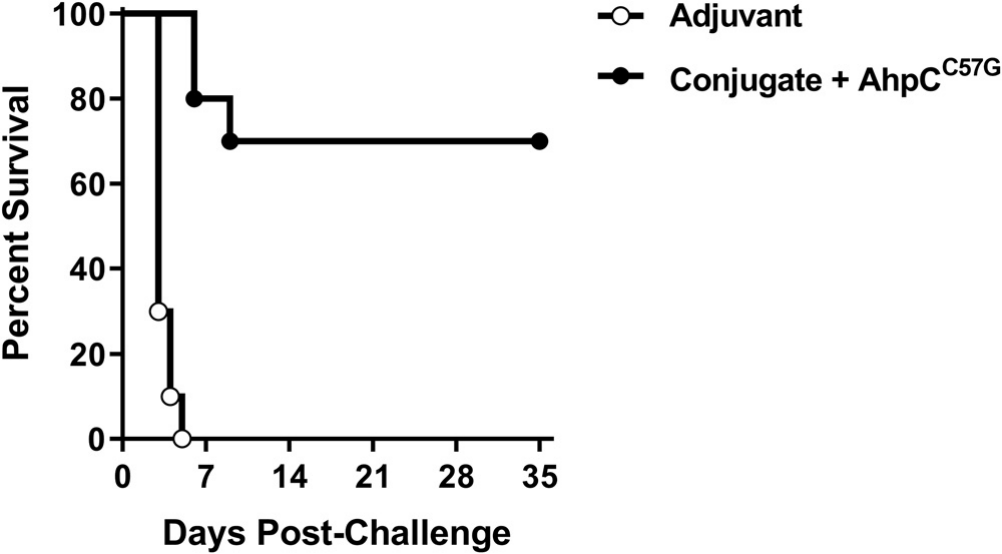
Protective capacity of CPS-CRM197 plus AhpC^C57G^. C57BL/6 mice (*n* = 10 per group) were immunized on days 0, 21, and 35 with adjuvant only or CPS-CRM197 plus AhpC^C57G^. Five weeks after the final boost, mice were challenged via an inhalational route with lethal doses of B. pseudomallei K96243. In the adjuvant-only group, 5 mice received 20 LD_50_s and 5 mice received 29 LD_50_s. In the conjugate-plus-AhpC^C57G^ group, 5 mice received 27 LD_50_s and 5 mice received 28 LD_50_s. Mice were then monitored for 35 days postchallenge, and their survival was plotted.

To determine the protective capacity of CPS-CRM197 plus AhpC^C57G^ in an animal model of melioidosis, immunized C57BL/6 mice were challenged with high inhalational doses (27 or 28 50% lethal doses [LD_50_s]) of B. pseudomallei K96243 5 weeks after the final boost and then monitored for signs of morbidity and mortality over a 35-day period. As expected, all the mice in the adjuvant-only group (challenged with 20 or 29 LD_50_s) rapidly succumbed to infection (≤5 days). In contrast, 70% of the mice immunized with AhpC^C57G^ plus CPS-CRM197 survived for the duration of the experiment (Fig. 6). This experiment demonstrates the vaccinogenic potential of AhpC^C57G^ in combination with CPS-CRM197 and suggests that inclusion of AhpC^C57G^ in future formulations warrants further investigation.

## DISCUSSION

The development of subunit vaccines represents a promising approach for immunization against melioidosis. These types of vaccines are antigenically defined and can be rationally designed to include protective antigens that have been identified through studies using human melioidosis samples and/or in animal models of melioidosis (28, 29, 36, 40–43). Based on current evidence, a vaccine that stimulates both humoral and cellular immune responses will be necessary for robust protection against melioidosis. Several studies have shown that the 6-deoxyheptan CPS expressed by B. pseudomallei is a key protective antigen that, when administered to mice as a glycoconjugate, stimulates high-titer IgG responses that are opsonizing (1, 19, 24). A number of protein antigens that correlate with protective T cell immunity in melioidosis patients have also been identified (28, 29, 40, 43). In previous studies, we have combined proteins such as Hcp1, TssM, and LolC with CPS-based glycoconjugates and assessed their potential as melioidosis vaccine candidates (19, 24). In this study, we admixed a CPS-CRM197 glycoconjugate with AhpC^C57G^ in the presence of Alhydrogel and CpG and demonstrated that this combination of antigens stimulated robust CPS- and AhpC^C57G^-specific antibody responses along with AhpC^C57G^-specific T cell responses in C57BL/6 mice. In addition, 70% of mice immunized with this formulation survived for 35 days following a high-dose aerosol challenge with B. pseudomallei.

The peroxiredoxin AhpC is an abundant, immunogenic protein that is considered a potential vaccine candidate for a variety of bacterial species, including Campylobacter jejuni, Helicobacter pylori, Bacillus anthracis, Mycobacterium tuberculosis, Streptococcus zooepidemicus, and Salmonella enterica serovar Typhimurium (44–48). B. pseudomallei AhpC has homology to the AhpC proteins from several of these organisms and harbors three conserved cysteine residues that are required for its function (31). AhpC is conserved across *Burkholderia* species, and in B. thailandensis, it has been demonstrated that AhpC plays a critical role in defense against oxidative stress (31). B. pseudomallei AhpC was identified as a serodominant antigen in a protein microarray study that assessed the reactivity with 747 individual serum samples from melioidosis patients and healthy donors from Singapore and Thailand. While AhpC was reactive with serum from melioidosis patients, it had low reactivity with serum from healthy donors (42). B. pseudomallei AhpC has also been shown to induce strong T cell-mediated responses in patients with acute melioidosis and was correlated with their survival (28, 29). Herein, we overexpressed B. pseudomallei AhpC in E. coli in soluble form and purified it in high yield to homogeneity using tandem nickel-cobalt chromatography. We also produced AhpC^C57G^, an enzymatically inactive version of the protein that would be considered safer for use as a vaccine candidate. His-tagged AhpC^C57G^ was isolated using the same procedure developed for His-tagged AhpC and resulted in high yields of pure protein.

Consistent with previous studies, our results demonstrated that B. pseudomallei AhpC is a highly immunogenic protein since immunization of mice with AhpC^C57G^ alone or in combination with CPS-CRM197 resulted in the production of robust antibody and/or mixed Th1-, Th2-, and Th17-like T cell responses (28, 29). Importantly, the antibodies raised against the recombinant AhpC^C57G^ recognized native AhpC in whole-cell lysates of B. thailandensis E555, which allowed for the assessment of the opsonizing capacity of both AhpC^C57G^- and CPS-specific antibodies in opsonophagocytosis assays. While CPS antibodies promoted uptake of B. thailandensis into RAW 264.7 cells, AhpC^C57G^ antibodies did not. These results confirmed the functionality of the CPS-specific antibodies generated against the formulation containing CPS-CRM197 combined with AhpC^C57G^. Peptide mapping studies also revealed the potential presence of both CD4^+^ and CD8^+^ T cell-restricted epitopes within AhpC^C57G^. Identification of peptides 2 and 12 was particularly useful for assessment of AhpC^C57G^-specific IFN-γ T cell responses via ELISpot assay. When stimulated with whole AhpC^C57G^ protein, the number of spot-forming cells (SFCs) in the adjuvant-only control mice was higher than anticipated. The reason for this phenomenon is unclear, but it is not inconsistent with ELISpot data obtained from T cell studies using human seronegative controls (29). Use of these peptides in the IFN-γ ELISpot assays resulted in reduced background levels in the adjuvant-only controls and thus will be useful for future studies focused on AhpC^C57G^ as a potential vaccine candidate. Additionally, peptides 2, 9, and 12 may be useful for helping to define correlates of immunity associated with this protein due to the three distinct cytokine patterns that they appear to stimulate (e.g., peptide 2 stimulates IFN-γ, IL-5, and IL-17, peptide 9 stimulates  IFN-γ and IL-17, and peptide 12 stimulates IFN-γ).

In a high-dose (27 to 28 LD_50_s) aerosol challenge study, 70% of the mice immunized with AhpC^C57G^ and CPS-CRM197 survived for 35 days following exposure to B. pseudomallei. While this is a promising result, this level of protection is below what has previously been observed using CPS-CRM197 in combination with Hcp1 (100%) or TssM (80%) (19). It should be noted, however, that those experiments were conducted with a significantly lower challenge dose (~10 LD_50_s) (19). Collectively, our results show that AhpC^C57G^ is a potent activator of cellular and humoral immune responses, and based on the outcome of the challenge studies, this warrants further investigation as a potential melioidosis vaccine candidate. Future studies examining the protective capacity of AhpC^C57G^ alone or in combination with existing CPS-CRM197-based vaccine formulations are under way.

## MATERIALS AND METHODS

### Bacterial strains, plasmids, and growth conditions.

The bacterial strains and plasmids used in this study are described in Table 1. E. coli strains with plasmids were cultured on Luria-Bertani-Lennox (LB; Fisherbrand) agar or in LB broth containing ampicillin (100 μg/mL). B. pseudomallei Bp82 was cultured in LB broth or on LB agar supplemented with thiamine (5 μg/mL) and adenine (100 μg/mL). B. thailandensis BT2683, B. thailandensis E555, and B. pseudomallei K96243 were cultured in LB broth or on LB agar. For Western immunoblotting experiments, B. pseudomallei Bp82 and B. thailandensis E555 were cultured in Difco M9 minimal salts supplemented with 0.4% glucose and 0.5% (wt/vol) Bacto Casamino Acids (M9CG; BD) with or without thiamine and adenine, respectively. All bacterial cultures were incubated at 37°C; broth cultures were incubated with shaking (200 rpm). Bacterial stocks were maintained at −80°C in 20% glycerol suspensions. All manipulations of B. pseudomallei K96243 were conducted in CDC/USDA-approved and registered BSL3 or animal biosafety level 3 (ABSL3) facilities at the University of Texas Medical Branch, and experiments were performed in compliance with the rules and regulations of the U.S. Federal Select Agent Program.

### Sequence alignment.

Protein sequences were aligned using CLUSTAL 2.1. The RefSeq accession numbers for sequences are as follows: WP_004192241.1 (Burkholderia pseudomallei K96243), WP_004192241.1 (Burkholderia mallei ATCC 23344), WP_004192241.1 (Burkholderia thailandensis E264), WP_034207560.1 (Burkholderia cepacia DM43), WP_003412529.1 (Mycobacterium tuberculosis H37Rv), WP_000052796.1 (Escherichia coli O157:H7), WP_023215210.1 (Salmonella enterica serovar Typhimurium SL1344), and WP_000961643.1 (Helicobacter pylori SS1).

### Glycoconjugate synthesis.

CPS was purified from B. thailandensis BT2683 using a modified hot aqueous-phenol procedure essentially as previously described (1, 27, 49). Recombinant, preclinical-grade CRM197 was purchased from Fina Biosolutions. The CPS-CRM197 glycoconjugates used in this study were synthesized as previously described (27). A bicinchoninic acid (BCA) assay kit (Pierce) was used to quantitate the protein concentration of the glycoconjugate stock. (The remainder of the mass was assumed to be polysaccharide.) The glycoconjugate material used in this study contained 60% (wt/wt) CPS.

### Protein expression and purification.

For expression of recombinant AhpC with an N-terminal 6×His tag, the *ahpC* open reading frame (ORF) (BPSL2096) was PCR amplified from B. pseudomallei K96243 genomic DNA using the BpAhpC-6HisF (5′-CCCAACCGTCTCCCATGGCGGCGCATCATCATCATCATCATAAGACCGTGGGCGATAAACTCGAAG-3′) and BpAhpC-R1 (5′-CCCAACCGTCTCTAGCTTTACAGCGTCGCGCCGCCGATC-3′) primer pair; BsmBI recognition sites are underlined. The resulting DNA fragment was digested with BsmBI and cloned into pBAD/HisA digested with NcoI/HindIII, producing plasmid pRR1000. For expression of recombinant AhpC^C57G^ with an N-terminal 6×His tag, a DNA fragment (*ahpC*-G57) in which the Cys codon (TGC) at position 57 in *ahpC* was changed to a Gly codon (GGC) was synthesized (GenScript) and then cloned into pCR2.1-TOPO to generate pLS1000. pLS1000 was digested with BsmBI to release the DNA fragment containing *ahpC*-G57 which was then cloned into pBAD/HisA digested with NcoI/HindIII to produce pLS1001. Recombinant DNA techniques were conducted as previously described (50). Oligonucleotide primers were obtained from Integrated DNA Technologies. DNA sequencing was performed by ACGT, Inc.

For purification of AhpC and AhpC^C57G^, E. coli TOP10(pRR1000, pLS1001) cells were grown to mid-log phase in LB broth, and protein expression was induced using 0.02% l-arabinose (Sigma). Bacterial pellets were resuspended in B-PER (Pierce) plus Benzonase (Novagen) and lysozyme (100 μg/mL) and incubated for 20 min at room temperature with gentle agitation. Insoluble material was removed by centrifugation, and the resulting supernatant was loaded onto a gravity-fed Ni-nitrilotriacetic acid (NTA) agarose (Invitrogen) column. The column was washed with wash buffer (40 mM Tris [pH 8.0], 300 mM NaCl, and 40 mM imidazole), and protein was eluted with elution buffer (40 mM Tris [pH 8.0], 50 mM NaCl, and 300 mM imidazole) and then dialyzed against phosphate-buffered saline (PBS). The resulting protein was then loaded onto a gravity-fed His-Pur cobalt resin (Thermo Scientific) column, washed with imidazole-free wash buffer (40 mM Tris [pH 8.0], 300 mM NaCl), eluted with elution buffer, and dialyzed against PBS. The AhpC and AhpC^C57G^ preparations used in peroxidase activity assays were purified in the presence of 0.25 mM TCEP [Tris(2-carboxyethyl)phosphine] (Thermo Scientific) to maintain them in a reduced state prior to dialysis into PBS. Recombinant Hcp1 harboring an N-terminal 6×His tag was purified from E. coli TOP10(pBADBmhcp1-6HisF), as previously described (37). Protein concentrations were determined using a BCA protein assay kit (Pierce). Endotoxin removal was performed using Pierce high-capacity endotoxin removal resin. The amount of endotoxin in the purified protein preparations was quantitated using a Pierce LAL chromogenic endotoxin quantitation kit as per the manufacturer’s instructions. Purified proteins were filter sterilized using a 0.45-μm-pore syringe filter and then stored at 4°C.

### SDS-PAGE.

Protein samples were mixed with an equal volume of 2× SDS-PAGE sample buffer and heated to 100°C for 10 min prior to electrophoresis on Bolt 12% Bis-Tris Plus gels (Invitrogen). Proteins were visualized using SimplyBlue SafeStain (Invitrogen).

### Peroxidase activity assay.

The enzymatic activities of AhpC and AhpC^C57G^ were determined indirectly by measuring the reduction of hydrogen peroxide using a quantitative peroxide assay kit (Pierce). This assay measures hydrogen peroxide levels in biological samples using an iron and xylenol orange reagent. In a 96-well plate (Costar), 170 ng H_2_O_2_ was combined with 20 μg of purified AhpC or AhpC^C57G^ and incubated for 10 min. To quantitate residual peroxide, the iron and xylenol orange reagent was prepared per the manufacturer’s instructions and added to each of the wells. The plate was incubated for 30 min at room temperature and then read at 595 nm using a FLUOstar Omega microplate reader (BMG Labtech). HRP (Sigma) and Hcp1 were used as positive and negative controls, respectively. Two independent experiments were performed in duplicate. To determine the maximal amount of H_2_O_2_ that could be reduced, the absorbance of 170 ng of H_2_O_2_ was determined in the absence of protein. Using the total H_2_O_2_ as a baseline, the amount of residual H_2_O_2_ in samples was calculated based on a change in the absorbance from the baseline.

### Ethics statement.

All investigations involving animals were conducted in strict accordance with the recommendations in the *Guide for the Care and Use of Laboratory Animals* (51). Protocols were approved by the Animal Care and Use Committees at the University of Nevada, Reno (protocol no. 00785), or the University of Texas Medical Branch (protocol no. 0503014D) as well as by the U.S. Army Medical Research and Development Command Animal Care and Use Review Office (protocol no. CB 2018-07.01 and CB 2018-07.02). Mice were housed in microisolator cages under pathogen-free conditions, provided with rodent feed and water *ad libitum*, and maintained on a 12-h light cycle.

### Mouse immunizations.

For peptide mapping studies, 6- to 8-week-old female C57BL/6 mice (*n* = 12) (Charles River Laboratories) were immunized subcutaneously on days 0, 21, and 35 with AhpC^C57G^ (5 μg/dose) formulated in PBS (pH 7.2) (Gibco) with Alhydrogel at 2% (500 μg/dose) (Brenntag) and CpG (20 μg/dose) (ODN 2006; InvivoGen) as the adjuvant system. One week after the final boost, spleens were harvested from terminally bled mice, and single-cell suspensions were prepared for use in ELISpot assays as described below.

For T cell depletion studies, 6- to 8-week-old female C57BL/6 mice (*n* = 9) (Charles River Laboratories) were immunized subcutaneously on days 0, 21, and 35 with AhpC^C57G^ (5 μg/dose) formulated in PBS (pH 7.2) (Gibco) with Alhydrogel at 2% (500 μg/dose) (Brenntag) and CpG (20 μg/dose) (ODN 2006; InvivoGen) as the adjuvant system. One week after the final boost, spleens were harvested from terminally bled mice, and single-cell suspensions were prepared for use in CD4^+^ and CD8^+^ T cell depletion experiments, as described below.

For the immunogenicity and challenge studies, 6- to 8-week-old female C57BL/6 mice (*n* = 16) (Charles River Laboratories) were immunized subcutaneously on days 0, 21, and 35 with CPS-CRM197 (2.5 μg/dose of CPS as a conjugate) plus AhpC^C57G^ (5 μg/dose) formulated in PBS (pH 7.2) (Gibco) with Alhydrogel at 2% (250 μg/dose) (Brenntag) and CpG (10 μg/dose) (ODN 2006; InvivoGen) as the adjuvant system. Mice (*n* = 16) immunized with adjuvant only served as the control group. One week after the final boost (day 42), serum and spleens were collected from terminally bled mice (*n* = 6 per group) and used in ELISpot assays, ELISAs, or opsonophagocytosis assays, as described below. The remaining mice in each group were used in challenge studies, as described below.

### AhpC peptide library.

The primary sequence of AhpC^C57G^ was used to design and synthesize a Think Peptides PEPscreen peptide library consisting of 35 peptides that were 15 amino acids in length and overlapped by 10 amino acids (ProImmune) (Table 2). Peptides were resuspended in dimethyl sulfoxide (DMSO) at a concentration of 10 μg/mL or 20 μg/mL (based on solubility) and stored at −20°C until required for use in ELISpot assays.

### ELISpot assays.

Spleens were harvested from immunized mice, and single-cell suspensions were prepared by passing the organs through 70-μm-pore cell strainers (Falcon) into RPMI 1640 (Gibco) medium supplemented with 10% (vol/vol) heat-inactivated (HI) fetal bovine serum (RPMI-10). Cells were pelleted by centrifugation (450 × *g*), resuspended in Sigma red blood cell lysis solution, incubated at room temperature for 10 min, pelleted (450 × *g*) again, and then resuspended in RPMI-10. Mouse IFN-γ, IL-5, and IL-17 ImmunoSpot ELISpot kits (Cellular Technology, Ltd.) were used per the manufacturer’s instructions. Splenocytes at a concentration of 2.5 × 10^5^ cells/well were stimulated with AhpC^C57G^ (2 μg/well), AhpC peptides (4 μg/well), or medium only for 48 h at 37°C under an atmosphere of 5% CO_2_. The ELISpot plates were processed and developed per the manufacturer’s instructions. Plates were imaged using an ImmunoSpot S6 analyzer (Cellular Technology, Ltd.). IFN-γ-, IL-5-, and IL-17-secreting T cells were quantitated using ImmunoSpot v7.0.22.0 Professional DC Smart Count software (Cellular Technology, Ltd.).

### CD4^+^ and CD8^+^ T cell depletion.

Spleens were harvested from immunized mice (*n* = 9), and single-cell suspensions were prepared by passing the organs through 70-μm-pore cell strainers into RPMI-10. Cells were pelleted by centrifugation (450 × *g*), resuspended in red blood cell lysis solution (Sigma), and then incubated at room temperature for 10 min. Cells were washed twice by being pelleted (450 × *g*) and then resuspended in RPMI-10, and then they were passed through a 40-μm-pore cell strainer (Falcon). Prior to treatment for T cell depletion, splenocytes prepared from 3 individual spleens were pooled, resulting in 3 groups of splenocytes. Each group was then split into 3 equal aliquots and individually processed for (i) CD4^+^ T cell depletion or (ii) CD8^+^ T cell depletion or (iii) were not depleted. CD4^+^ or CD8^+^ T cells were depleted by positive selection using magnetic antibody cell separation (MACS) CD4 (L3T4) MicroBeads and CD8a (LY-2) MicroBeads with LD separation columns (Miltenyi) per the manufacturer’s instructions. The undepleted samples (not treated with MicroBeads) were run on MACS LD separation columns for control purposes. The column eluates were pelleted by centrifugation (450 × *g*) and then resuspended in RPMI-10. Mouse IFN-γ ELISpot assays were conducted as described above.

### Analysis of antibody titers.

Terminal bleeds were conducted on immunized mice (*n* = 6 per group) 1 week after the final boost. Serum was processed using Vacutainer SST tubes (BD) per the manufacturer’s instructions and stored at −80°C until required for use. Antibody responses directed against the vaccine antigens were assessed by ELISAs as previously described. Briefly, 96-well Maxisorp plates (Nunc) were coated overnight at 4°C with purified AhpC^C57G^ or CPS (1 μg/mL) solubilized in carbonate buffer (pH 9.6). The plates were blocked at room temperature for 30 min with StartingBlock T20 (Tris-buffered saline [TBS]) blocking buffer (Thermo Scientific) and then incubated for 1 h at 37°C with mouse serum samples serially diluted in TBS plus 0.05% Tween 20 (TBST; pH 7.5) plus 10% StartingBlock T20. To facilitate detection, the plates were incubated for 1 h at 37°C with 1/2,000 dilutions of anti-mouse IgM, IgG, IgG1, IgG2b, or IgG2c HRP-conjugated antibodies (Southern Biotech). The plates were developed with TMB (3,3′,5,5′-tetramethylbenzidine) substrate (KPL) and read at 620 nm using a FLUOstar Omega microplate reader (BMG Labtech). The reciprocals of the highest dilutions exhibiting optical densities (ODs) that were 3× background levels were used to determine the endpoint titers for the individual mice.

### Western immunoblotting.

B. pseudomallei Bp82 and B. thailandensis E555 whole-cell lysates were separated on Bolt 12% Bis-Tris Plus gels and electrophoretically transferred to nitrocellulose membranes. The membranes were blocked with StartingBlock T20 (TBS) blocking buffer for 1 h at room temperature and then incubated for 1 h at room temperature with a 1/1,000 dilution of pooled serum from immunized mice. To facilitate detection, the membranes were incubated for 1 h at room temperature with 1/5,000 dilutions of anti-mouse IgG HRP-conjugated antibodies (Southern Biotech). Blots were visualized using Pierce ECL Western blotting substrate (Thermo Scientific) and a ChemiDoc imaging system (Bio-Rad).

### Opsonophagocytosis assays.

Opsonophagocytosis assays were conducted essentially as previously described (19). Briefly, B. thailandensis E555 cultures grown to early log phase were pelleted, resuspended at a density of 1 × 10^6^ CFU/mL in Dulbecco’s modified Eagle’s medium (DMEM) or DMEM containing 1% adjuvant only, AhpC^C57G^, or CPS-CRM197 plus AhpC^C57G^ mouse immune serum (pooled and heat inactivated for 30 min at 56°C) and incubated at 37°C for 1 h. The opsonized bacterial suspensions were added to RAW 264.7 cells (1 × 10^6^ cells/well) and incubated at 37°C under an atmosphere of 5% CO_2_ for 1 h. Infected monolayers were then washed twice with Hanks’ balanced salt solution (HBSS) (Gibco) and incubated with fresh DMEM-10 containing 250 μg/mL kanamycin for 2 h. Following this, the infected monolayers were washed twice with HBSS and lysed with 0.2% (vol/vol) Triton X-100 (Sigma), and serial dilutions of the lysates were plated onto LB agar plates and incubated at 37°C for approximately 48 h. Plate counts were used to enumerate bacterial loads. Three independent experiments were performed in duplicate.

### Mouse challenge studies.

Five weeks after the final boost (day 70), control and immunized mice (*n* = 10 per group) were challenged via an inhalational route with B. pseudomallei K96243, as previously described (19, 52). Briefly, groups of mice were exposed to aerosolized bacteria at nebulizer concentrations of ~4.48 × 10^7^ to 4.96 × 10^7^ CFU/mL for 15 min at a constant flow rate of 30 L/min. Doses presented (Dp) to each group of animals were determined by performing standard CFU counts on the samples and then using the following formulas: (i) Dp (CFU) = C_Aero_ (CFU/mL) × exposure time (min) × min vol (mL) and (ii) min vol = 2.1(wt [g])^0.75^. In the adjuvant-only group, 5 mice received 20 LD_50_ (Dp = 2,940 CFU) and 5 mice received 29 LD_50_ (Dp = 4,340 CFU). In the conjugate-plus-AhpC^C57G^ group, 5 mice received 27 LD_50_s (Dp = 4,040 CFU) and 5 mice received 28 LD_50_s (Dp = 4,180 CFU). Weight and survival of the challenged mice were monitored for 35 days. Humane endpoints were strictly observed via daily monitoring throughout the study.

### Statistical analysis.

All graphs were produced by using GraphPad Prism 8.2.0 (GraphPad Software, Inc.). Opsonophagocytosis and ELISpot assay data were analyzed using a Mann-Whitney U test. Survival data were analyzed using a log-rank (Mantel-Cox) test.
